# Unveiling the egg microbiota of the loggerhead sea turtle *Caretta caretta* in nesting beaches of the Mediterranean Sea

**DOI:** 10.1371/journal.pone.0268345

**Published:** 2022-05-26

**Authors:** Luca Vecchioni, Antonino Pace, Arianna Sucato, Flavia Berlinghieri, Irene Cambera, Giulia Visconti, Sandra Hochscheid, Marco Arculeo, Rosa Alduina

**Affiliations:** 1 Department of Biological, Chemical and Pharmaceutical Sciences and Technologies (STEBICEF), University of Palermo, Palermo, Italy; 2 Stazione Zoologica Anton Dohrn, Department of Marine Animal Conservation and Public Engagement, Marine Turtle Research Group, Portici (NA), Italy; 3 Groningen Institute for Evolutionary Life Sciences, University of Groningen, Groningen, The Netherlands; 4 Department of Biological Sciences, Macquarie University, Sydney, Australia; 5 Pelagie Islands Marine Protected Area, Municipality of Lampedusa and Linosa, Agrigento, Italy; Universita degli Studi della Tuscia, ITALY

## Abstract

Microbes have central roles in the development and health of animals, being the introduction of specific microbial species a potential conservation strategy to protect animals from emerging diseases. Thus, insight into the microbiota of the species and their habitats is essential. In this manuscript, we report for the first time the bacterial composition of all the components (eggshells of hatched and unhatched eggs, internal content of unhatched eggs, intestinal content of hatchling and pipping sea turtles, and sand) of three nesting beaches of *Caretta caretta* along the Italian coasts of the Mediterranean Sea. The analysis of 26 amplicon samples was carried out using next-generation sequencing analysis, targeting V3–V4 regions of the bacterial 16S rRNA gene. Samples featured mainly Proteobacteria, Actinobacteria, Firmicutes, and Bacteroidetes, whose percentages depended on the sample type. Our results showed that, although from different sampling sites, the internal content of the unhatched eggs, intestinal content of hatchling and pipping sea turtles share the microbiota, which was yet different from that of eggshells and sand of the same nesting beach. This study suggests the maternal and environmental influence alongside a protective role of eggshells in shaping the egg microbiota of *Caretta caretta* sea turtles.

## Introduction

*Caretta caretta* L. is the most common sea turtle species nesting along the coasts of the Mediterranean Sea. It is currently considered “Least Concern” by IUCN [1] https://www.iucnredlist.org/species/3897/119333622 even though incidental fisheries bycatch [2], water and sand pollution, pathogens [3–6], global climatic changes [7–10], and low hatching success [11] represent real threats. For example, the latter depends on multiple factors, including genetic and environmental ones, as previously defined [11,12].

The microbiota is inherited in many vertebrates [13,14], and host phylogeny might affect its structure and role [15,16], with co-evolution of the host and microbiota being an essential relationship in modelling metazoan life [17–19]. Although several reports described the gut, cloacal, oral and carapace microbiota of the Mediterranean loggerhead sea turtles [20–24], data on the bacterial flora of *C*. *caretta* nests are scarce.

*C*. *caretta*, like most reptiles, is an oviparous species without parental care. However, the maternal bacterial flora could have a role in shaping egg microbiota during the permanence in the uterine tube, the passage through the cloaca, or the deposition, as shown in other reptiles [14,25–27]. Besides, hatchlings could share the microbiota with the nesting beaches where the females lay eggs [28], even though hatchlings do not consume specific excrements or ingest soil from areas surrounding the nest. These phenomena feature other animals that do not take advantage of maternal care, such as marine and green iguanas, to jump-start a healthy gut microflora [29,30].

Up to date, a few studies describe microbes, mainly pathogens, associated with unhatched sea turtle eggs [31–34], whose investigation mostly relies on classical microbiological methods, searching for pathogens of both bacterial and fungal origin [35]. Since most bacteria do not grow on standard culture media and under laboratory conditions, these methods under-represent the whole microbial flora. Nowadays, next-generation sequencing of 16S rDNA from an amplicon sample is considered a quick, simple, and inexpensive method to unveil the bacterial community [36–39]

**Fig 1:**
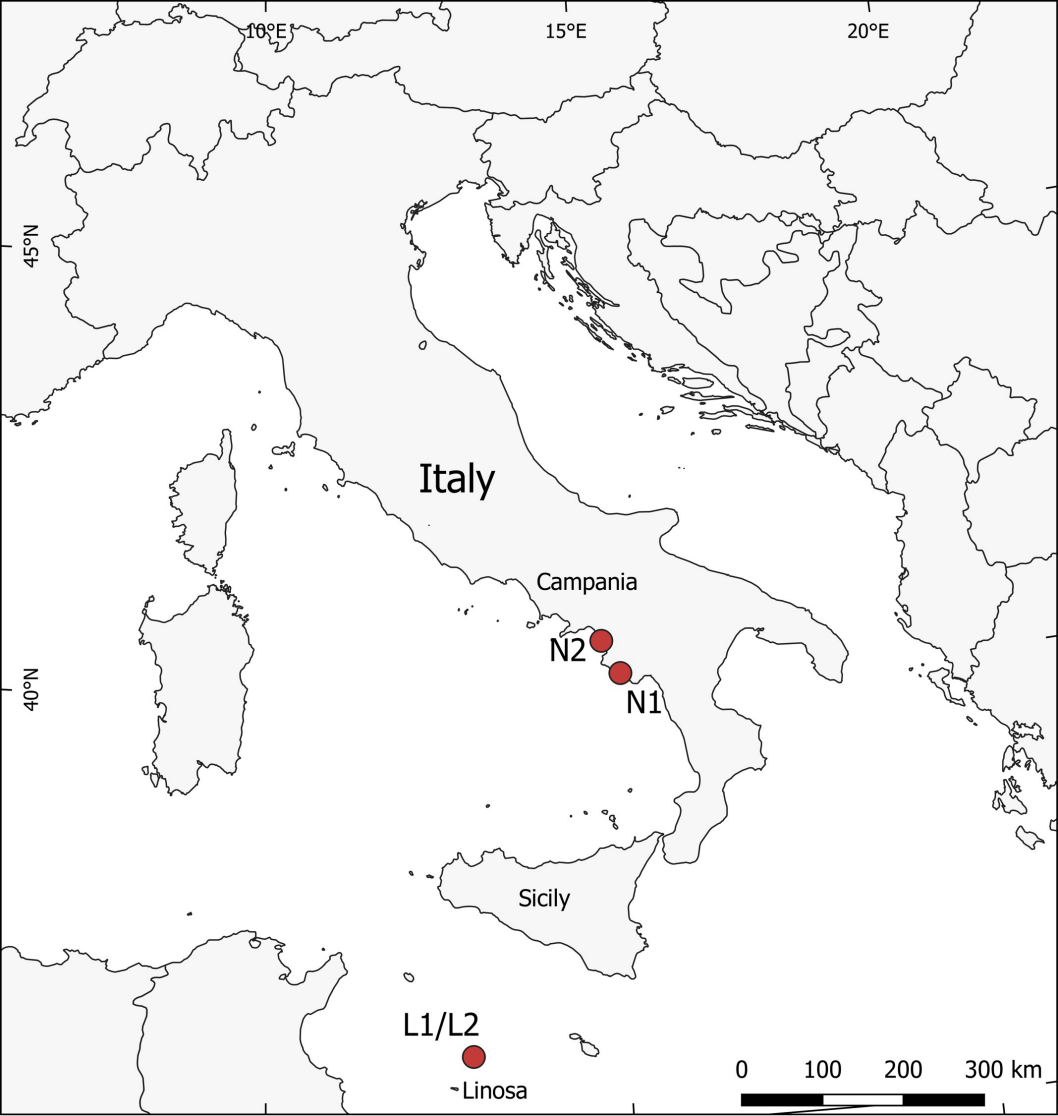
Map of the nesting beaches. L1 and L2, Linosa; N1, Ascea Marina; N2, Eboli.

This study describes, for the first time, the microbiota of eggshells of hatched and unhatched eggs, the internal content of unhatched eggs, the intestinal one of hatchling/pipping sea turtles, and the sand of four nests of the loggerhead sea turtles *C*. *caretta*, deriving from three nesting beaches of the Mediterranean Sea (Fig 1).

## Materials and methods

### Sample collection

The sampling was carried out in two areas of the Mediterranean Sea: Pozzolana di Ponente beach at Linosa island within Pelagie Islands Marine Protected Area (Strait of Sicily, Italy) during two campaigns (2018 and 2019), and two beaches, Ascea Marina and Eboli, in Campania (Tyrrhenian coasts, Italy) in 2019. The map of the sampling sites was created using the QGIS software v. 3.18.3 [40] using the layer “ne_10m_admin_0_scale_rank_minor_islands.shp” freely available at www.naturalearthdata.com/downloads/.

About 72 hours after the last emerged hatchling from the nest chambers, the nest was dug to examine and catalogue the nest contents (eggshells, unhatched eggs, dead or alive hatchling and pipping). Fragments of eggshells, unhatched eggs, dead hatchlings and pipping specimens of loggerhead sea turtle *C*. *caretta* and sand were collected in separate sterile bags, following indications previously reported [41]. Sand samples were collected at a depth of about 20 (Top) and 50 (Bottom) cm of the nesting chamber. All samples were stored immediately at -20°C until DNA extraction. Details of the samples are listed in Table 1 and the nesting beaches are indicated in Fig 1 (Linosa (1–2): 35.863277 N, 12.854687 E; Eboli: 40.504083 N, 14.929983 E; Ascea Marina: 40.126240 N, 15.180160 E).

**Table 1:** Description and diversity indices of the samples used in this study.

Type	Sample	Brief description	Location	Year	S	Good’s coverage	Chao1	ACE	α	1-D	H’	e
Eggshell	ES_L1_1	Eggshell fragments	Linosa (Sicily)	2018	84	0.99	227.27	219.03	3.69	0.03	3.78	0.85
	ES_L2_1		Linosa (Sicily)	2019	56	0.99	78.63	85.18	2.00	0.02	3.69	0.91
	ES_L2_2		Linosa (Sicily)	2019	63	0.99	102.67	108.44	2.26	0.03	3.67	0.88
	ES_L1_2	Unhatched egg	Linosa (Sicily)	2018	57	0.99	232.87	232.28	2.91	0.03	3.64	0.90
	ES_L2_3		Linosa (Sicily)	2019	59	0.99	165.89	160.35	2.16	0.03	3.70	0.90
	ES_N1_1		Ascea Marina (Campania)	2019	36	0.99	174.36	167.77	2.66	0.09	2.88	0.80
	ES_N1_2		Ascea Marina (Campania)	2019	59	0.99	182.45	176.34	3.23	0.05	3.37	0.82
	ES_N2_3		Eboli (Campania)	2019	49	0.99	193.14	185.31	2.69	0.03	3.51	0.90
	ES_N2_4		Eboli (Campania)	2019	42	0.99	197.77	190.91	2.33	0.03	3.40	0.91
Internal Content	The stomach	Stomach of a hatchling turtle	Linosa (Sicily)	2018	32	0.99	232.71	228.37	2.06	0.05	3.10	0.89
	IC_L2_2		Linosa (Sicily)	2019	16	1	138.09	137.24	1.56	0.06	2.54	0.91
	IC_N1_3		Ascea Marina (Campania)	2019	34	0.99	221.40	214.17	2.05	0.04	3.10	0.89
	IC_N2_2		Eboli (Campania)	2019	11	0.99	218.93	211.61	2.90	0.18	1.90	0.79
	The stomach	Stomach of a pipping turtle	Linosa (Sicily)	2018	30	0.99	231.52	226.61	1.93	0.06	3.01	0.88
	IC_L2_1		Linosa (Sicily)	2019	30	0.99	122.98	125.57	1.96	0.04	3.13	0.92
	IC_N1_1		Ascea Marina (Campania)	2019	24	0.99	216.44	209.48	2.12	0.04	2.99	0.94
	The yolk1_3	Yolk of an unhatched egg	Linosa (Sicily)	2018	40	0.99	232.87	231.24	1.87	0.03	3.38	0.91
	IC_L2_3		Linosa (Sicily)	2019	55	0.99	153.90	151.29	2.07	0.02	3.71	0.92
	IC_N1_5		Ascea Marina (Campania)	2019	30	0.99	227.73	218.77	1.96	0.04	3.09	0.90
	IC_N2_4		Eboli (Campania)	2019	31	0.99	224.97	215.82	2.00	0.03	3.18	0.92
Sand	Sn_L1_1	Top	Linosa (Sicily)	2018	86	0.98	229.99	221.70	3.69	0.09	3.44	0.77
	Sn_N1_2		Ascea Marina (Campania)	2019	78	0.98	210.67	199.61	4.03	0.05	3.58	0.82
	Sn_N2_4		Eboli (Campania)	2019	87	0.99	215.00	207.76	3.32	0.04	3.77	0.84
	Sn_L1_2	Bottom	Linosa (Sicily)	2018	158	0.98	229.25	223.12	5.55	0.16	3.40	0.67
	Sn_N1_1		Ascea Marina (Campania)	2019	89	0.99	204.75	195.24	4.60	0.12	3.32	0.74
	Sn_N2_3		Eboli (Campania)	2019	12	0.96	212.53	204.16	27.91	0.90	0.30	0.12

Eggshell (ES) indicates the eggshells of the hatched and the unhatched eggs whereas internal content (IC) indicates the intestinal content of hatchling and pipping sea turtles and the internal content of the unhatched eggs (yolk). Sand (Sn) indicates the collected sand samples in the nest; top and bottom refer to sand samples collected at a depth of 20 and 50 cm, respectively. Location (L1 and L2, Linosa; N1, Ascea Marina; N2, Eboli) and year of the sampling are reported. The numbers shown after the location codes, i.e., L1, L2, N1 and N2, represent the sample numbers. S represents the total number of bacterial families; Chao1 and ACE are abundance-based richness estimators; α is the alpha diversity; 1-D is the Simpson’s index; H’ is the Shannon-Wiener diversity; e is the evenness.

Collection of the samples was done under permission of the Italian Ministry of Environment and ISPRA (based on national law DPR 357/97) for MPA Pelagie Islands (n. 17054 del 20/07/2018/MATTM) and Ascea Marina and Eboli (n. 0024471/PNM 22/11/2016).

### Genomic DNA extraction, PCR amplification and sequencing

The external surface of the unhatched eggs was washed with ethanol for three minutes, the eggshell was punctured using a pipette tip, and the egg content was transferred into sterile tubes. Hatchling and pipping sea turtles were dissected to collect the gastrointestinal tract that was transferred into sterile tubes. DNA extraction was carried out using 1 g of sand, yolk egg, the gastrointestinal tract of hatchling and pipping sea turtles and small fragments of the eggshells (both from hatched and unhatched eggs), following the protocols previously reported [22] with minor modifications. Specifically, the samples of sand, yolk egg and the gastrointestinal tract were incubated in 1 ml of sterile water for 1 h at room temperature (500 rpm). The small fragments of the eggshells (of the same size as a 50 ml tube stopper) were homogenized by vortexing in 3 ml of sterile water using sterile glass beads and stirred for 1 h at room temperature. Following this incubation step, samples were processed similarly. After the addition of 10 mg of lysozyme (Sigma-Aldrich) per ml of sample, the samples were further incubated at 37°C for 1 h. 0.1 mg/ml Proteinase K and 3% SDS were added and the samples were incubated at 55°C for 90 minutes. After incubation, the eggshells’ fragments were removed, and the solution was used to extract the total DNA. 2 ml of 5 M NaCl were added and samples were mixed by inversion. After the addition of 5 ml of chloroform, the samples were mixed by inversion for 30 minutes at room temperature. Samples were then centrifuged at 4500×g for 15 minutes at 4°C. The supernatant was transferred to a fresh tube and 0.6 volumes of isopropanol were added. Samples were then centrifuged at 13000 ×g for 30 minutes at 4°C. The supernatant was aspirated and discarded and the DNA pellet was washed several times with 70% ethanol and resuspended in 1 ml di TE (10 mM Tris-Cl, pH 8.0, 1 mM EDTA). The purity and quantity of DNA were assessed using Nanodrop (Thermo Fisher Scientific, Waltham, MA). An equal amount of the extracted DNA from each sample was sent to BMR Genomics for DNA sequencing of the V3-V4 region of the 16S rDNA using the primers previously described in [42] Pro341F: 5’-CCTACGGGNBGCASCAG -3’ and Pro805R: Rev 5’-GACTACNVGGGTATCTAATCC -3’ in one 300-bp paired-end run on an Illumina MiSeq platform. Unfortunately, PCR products from the sand of the L2 nest were not obtained.

### Raw data processing and statistical analyses

QIIME2 pipeline ([43], https://qiime2.org –build number 2021.4) was used starting from the paired-end sequences. In addition, the plug-in DADA2 was used to demultiplex, remove chimaeras and denoise all sequences, obtaining the ASV (Amplicon Sequence Variants).

Taxonomy was assigned using the SINA classifier on the “SILVA” [44] website (available at https://www.arb-silva.de/ngs/ - release 138.1). Rarefaction analysis was carried out by plotting the number of the observed ASVs against the total number of filtered reads for each sample.

The samples collected from the intestinal content of the four hatchlings, three pipping sea turtles, and four yolks of unhatched eggs were grouped and are indicated as internal contents, IC. Similarly, the microbiota of the eggshells of both hatched and unhatched eggs were grouped and are indicated as ES. Two-way ANOVA (Analysis of Variance) was made with the statistical software Minitab v. 17, testing as a null hypothesis the absence of a significant difference among different types of samples’ origin (“Type”, a fixed and orthogonal factor that includes three levels: eggshell, EG, sand, Sn, internal content of the eggs, IC), and sites of nesting beaches’ collection (“nesting beach”, fixed and orthogonal factor that includes four levels, i.e., both sites of Sicily and Campania) among all the detected families. Principal Coordinates Analysis (PCoA) was performed starting from the Bray–Curtis distance matrix, previously transformed using the square root, using the software package PRIMER 6 [45]. Two Expression Heat Maps were implemented through the online webserver (http://heatmapper.ca/expression/) [46] based on all detected classes and families. Both heat maps were generated by a “complete linkage” calculation using the Spearman Rank correlation.

Alpha diversity, Abundance-based Coverage Estimator (ACE), Chao1, Shannon-Wiener diversity, H’, and Simpson index, 1-D, and evenness, e, were estimated to determine the specific microbial richness and diversity. Good’s coverage was estimated to evaluate the completeness of sampling. The sequence dataset was deposited in the GenBank database (BioProject ID: PRJNA739563).

## Results

### Sequencing output and analysis

In total, 1269957 high-quality reads (Q*>*33 and 410 bp in size) were filtered from 1682981 raw reads obtained (S1 Table). 4557 unique ASVs were successfully identified using the QIIME2 pipeline and classified at the family level using a 97% sequence similarity threshold against the “SILVA” database. Excluding the unclassified families, 38 phyla, 78 classes, 164 orders and 232 families were detected.

A mean of 70.6 ±29.6 families was obtained from sand samples, 53 ±10.6 from eggshells, and a reduced number from internal content (28.72 ±11.2).

Rarefaction curves showed a good level of diversity sampling, as confirmed by Good’s coverage index for all the samples with an average of 0.99 (Table 1 and S1 Fig). Furthermore, the Shannon-Wiener diversity index was on average 3.29 ±0.9. The Simpson index ranged between 0.02 and 0.9 while evenness ranged between 0.12 and 0.94 (Table 1). Only the sample Sn_N2_3 showed a very low diversity.

Considering three types of samples (sand, eggshells and internal content) and four nesting beaches (L1, L2, N1 and N2), the two-way ANOVA was considered based on the detected families. A significant statistical difference among microbial composition dependent upon the sample type (*p* <0.001) was highlighted. Conversely, no significant differences were revealed among the four nest chambers (Table 2).

**Table 2:** Two-way ANOVA demonstrated that type of sample affected microbial diversity.

Source	DF	Adj SS	Adj MS	F-value	*p*-value
Nesting beach	3	1142.1	380.7	1.42	0.267
Type of sample	2	7823.2	3911.6	14.56	**0.000**
Error	20	5371.6	268.6		
Lack-of-Fit	5	690.9	138.2	0.44	0.812
Pure Error	15	4680.7	312.0		
Total	25	13822.3			

DF: Total degrees of freedom; Adj. SS: Adjusted sums of squares; Adj. MS: Adjusted mean squares. Bolded *p*-value was significant (*p* < 0.05).

### Distribution of microbiota diversity

**Fig 2:**
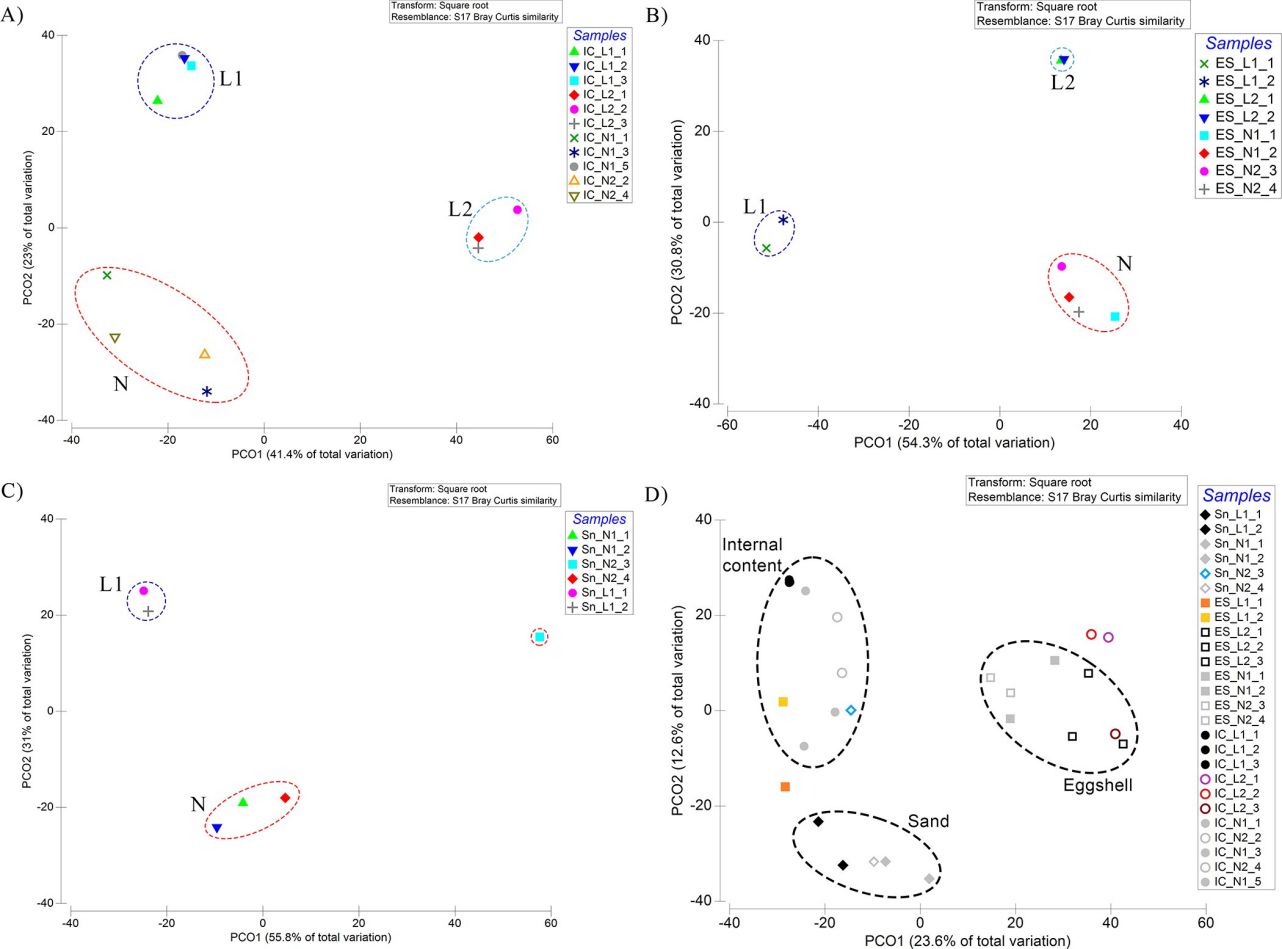
Principal Coordinates Analysis (PCoA) plot of IC (A), ES (B), and Sn (C) analysed in this study. D) PCoA plot of all the samples of this study.

The PCoA of the microbiota of the IC showed three clusters, comprising the microbiota of the two nests L1 and L2 and those of the nests N1+N2, which were very close (Fig 2A). Similarly, we obtained the same result when PCoA of the microbiota of the eggshells was determined. Our analysis demonstrated three clusters, comprised of L1, L2 and N1+N2 together (Fig 2B). PCoA of the microbiota of sand samples showed two clusters, one containing L1 sand, and the second one both the N1 and N2 sand samples (Fig 2C). The distance of the sample collected from the bottom sand of Campania nest 2 (Sn_N2_3) was expected, as the diversity indices have already indicated (Table 1). PCoA of the microbiota of IC, ES, and sand showed three well-defined clusters. The first cluster contained the microbiota of the internal contents of N1, N2 and L1 (top left Fig 2D), the second one the samples collected from the L2, N1 and N2 eggshells (right Fig 2D), a third one comprising all the sand samples N1, N2 and L1 except Sn_N2_3 (bottom Fig 2D). All the samples collected from the IC_L2 did not group with similar samples but with ES samples and the two ES_L1 samples were also outliers with respect to their group type.

**Fig 3:**
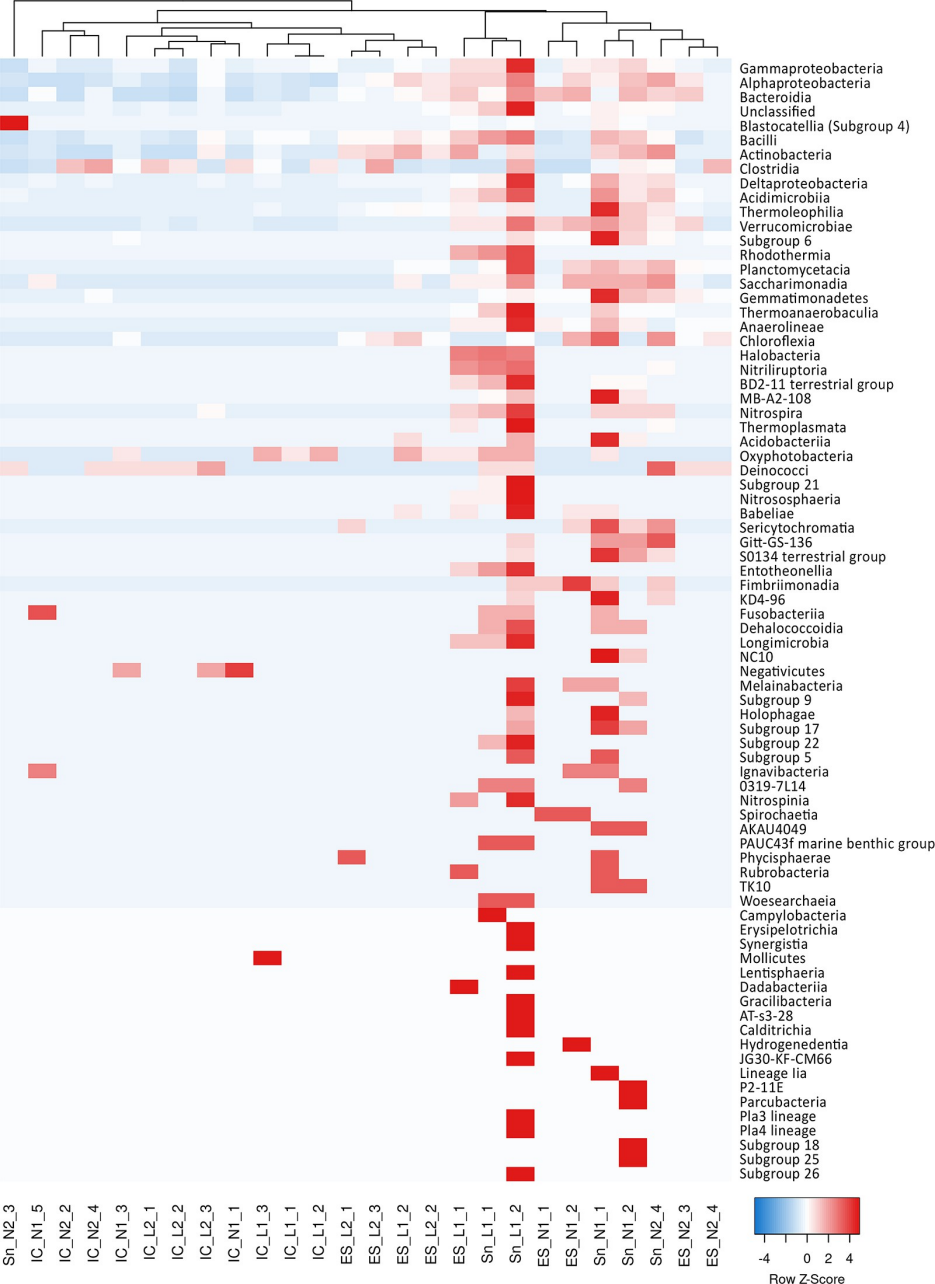
Heat Map based on the detected classes of all studied samples, generated by the “complete linkage” calculation and using Spearman Rank correlation.

A heat map profile was constructed to investigate the difference in microbial abundance between the samples. The heatmap of bacterial phyla showed two groups, one corresponding to all the IC samples and the other one to all the ES and Sn samples, that were similar to each other. Only Sn_N2_3 and ES_L2_3 samples were strangely grouped (S2 Fig). When the heatmap analysis was performed on bacterial classes (Fig 3), four groups were formed, one contained all the samples from L2, except IC_L2_2, the second one contained all the eggshells samples collected from N1 and N2, the third one the sand and the IC samples from N1 and N2, the fourth all the samples from L1. Although four samples fall outside of these four groups, this result strongly suggests the importance of the environment in which the eggs are laid.

### Microbial composition of sand samples

**Fig 4:**
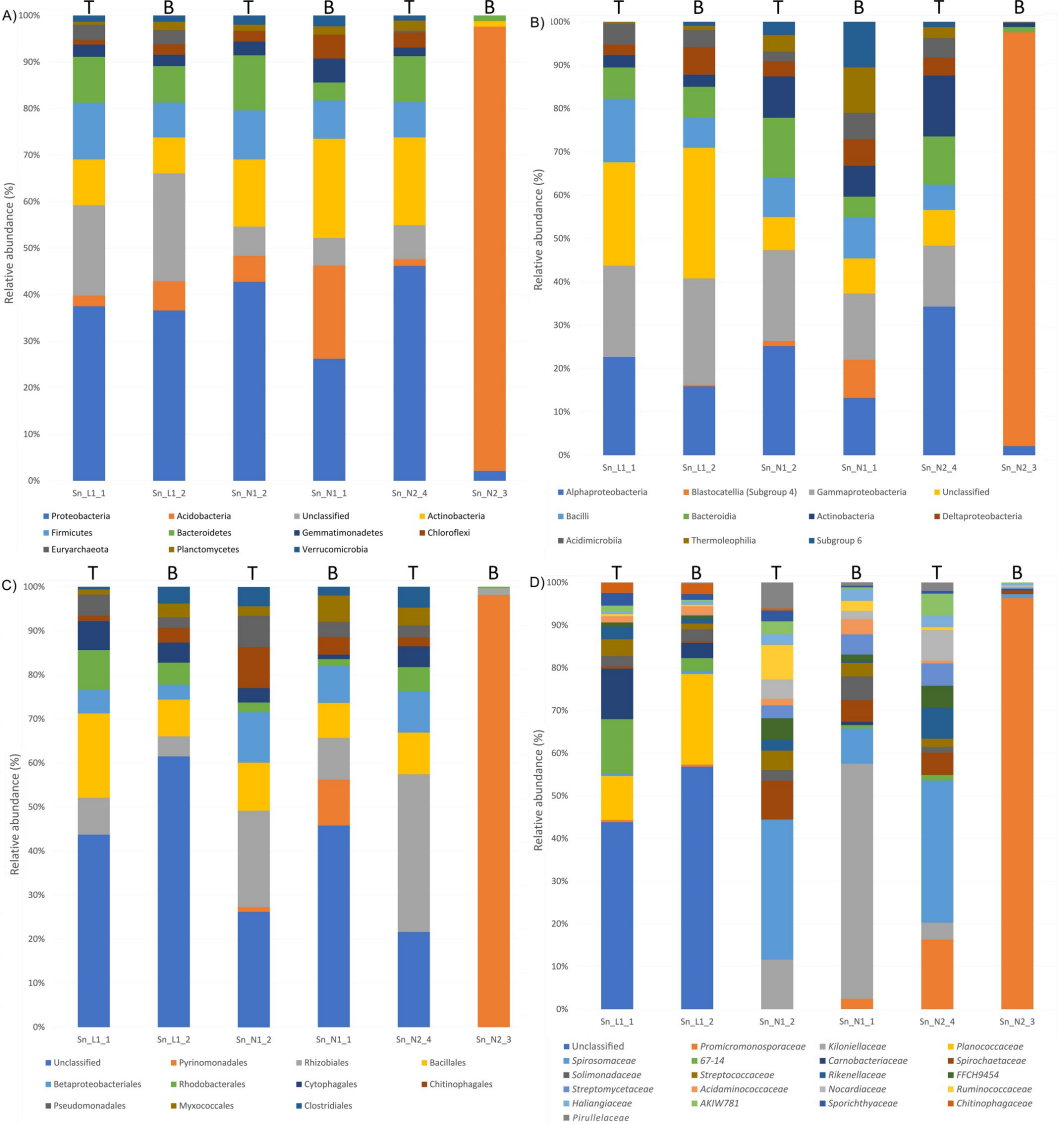
Percentage of the main 10 phyla (A), 10 classes (B), 10 orders (C) and 20 families (D) found in six samples of sand, collected from Linosa (indicated as Sn_L1), Ascea Marina (indicated as Sn_N1) and Eboli (indicated as Sn_N2) nests. For each nesting chamber, a sample was collected 20 cm below (indicated with a T, i.e., Top) and the other one 50 cm below the ground (indicated with a B, i.e., Bottom).

At the phyla level, the sand microbial composition was quite similar among all the samples (Fig 4A), except sample Sn_N2_3, which resulted extraordinarily enriched in Acidobacteria (95.5%). Although the low diversity of this sample, we decided to keep it since Acidobacteria were also present and more abundant in the other two bottom sand samples (Sn_N1_1 and Sn_L1_2), thus we cannot surmise whether this result was due to a technical issue or aberrant microbial colonization of the nest. The sand of Linosa was enriched with Proteobacteria (approximately 37%), Firmicutes (12.2 and 7.5%), Bacteroidetes (9.9 and 7.9%), Actinobacteria (9.9 and 7.7%), Acidobacteria (2.3 and 6.3%), Euryarchaeota (3.3 and 3%), and Gemmatimonadetes (2.6 and 2.4%). Three out of four sand samples of Campania (excluding that one with a low microbial diversity Sn_N2_3) were quite similar and were characterized by more Proteobacteria in two cases (42.8 and 46.2%) and less in one nest (2.1%) and more Actinobacteria (21.3, 14.5, and 18.9%) than Linosa nesting beach. Campania nests contained fewer Bacteroidetes in two cases (3.9 and 1.2%). Acidobacteria were highly abundant in two nests of Campania (95.5 and 20.1%) and less represented in the other two (5.6 and 1.5%) similarly to the Linosa nest. Gemmatimonadetes (5.1%) and Chloroflexi (5.1%) were more abundant in one nest of Campania. In one Campania nest, Euryarchaeota were very low in one sand sample (0.4%) and absent in the other three. Interestingly, a higher number of sequences of unknown bacteria was detected in the Linosa nesting beach. Analysis of bacterial families (Fig 4D), *Carnobacteriaceae*, *67–14* and *Planococcaceae* families resulted more abundant in the Linosa nest 1, *Kiloniellaceae*, *Spirosomaceae* and *Spirochaetaceae*, in Campania nest 1 and *Promicromonosporaceae* and *Spirosomaceae* in Campania nest 2 (Fig 4D). This result suggests specific microbial signatures on each beach, that could influence the microbial egg composition.

### Microbial composition of the eggshells

**Fig 5:**
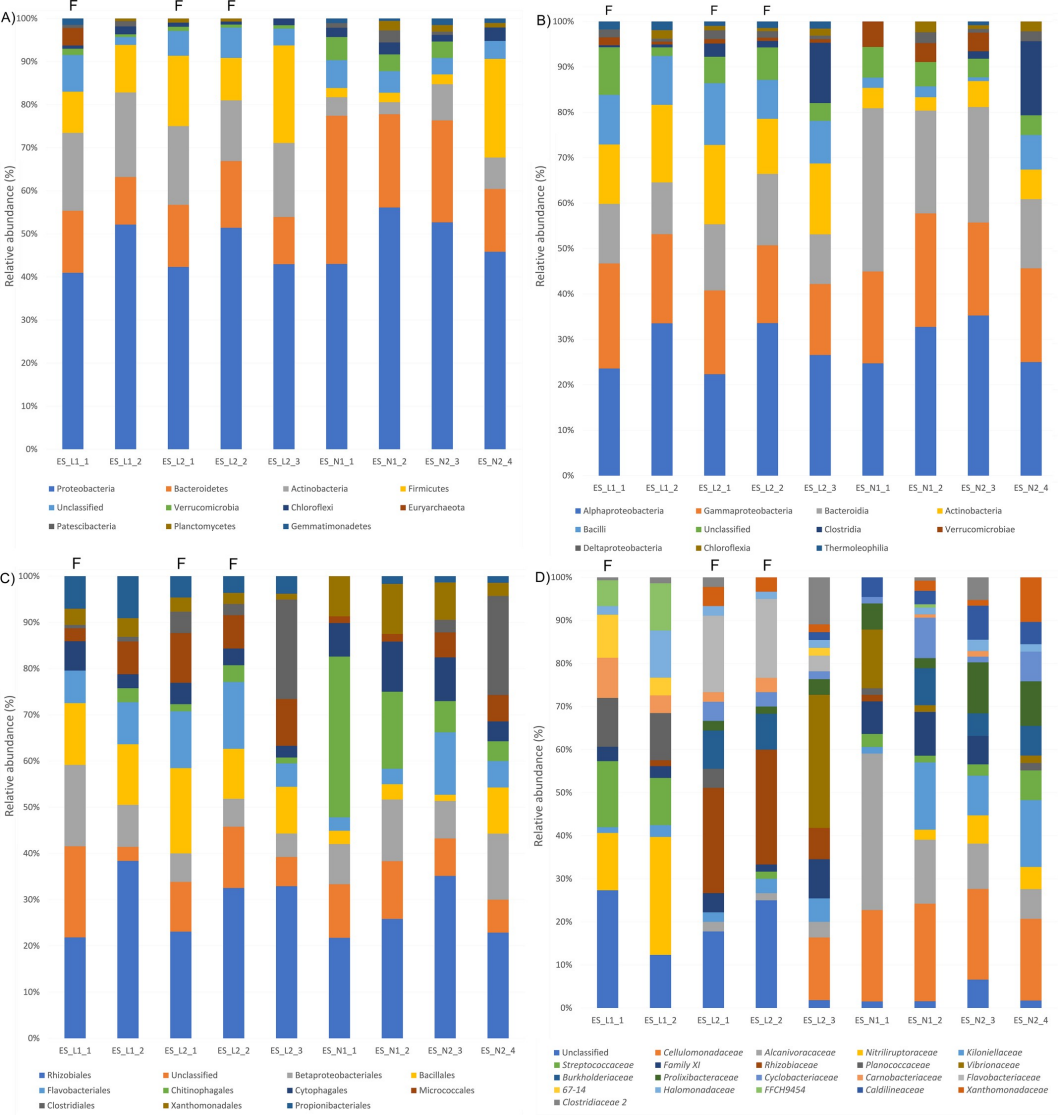
Percentage of the main 10 phyla (A), 10 classes (B), 10 orders (C) and 20 families (D) found in six samples of eggshells, collected from Linosa 2018 (indicated as ES_L1), Linosa 2019 (indicated as ES_L2), Ascea Marina (indicated as ES_N1) and Eboli (indicated as ES_N2) nests.

Eggshells were found to be abundant in the phyla Proteobacteria, Bacteroidetes, Actinobacteria and Firmicutes (Fig 5A). The main difference between Linosa and Campania nests was the abundance of Actinobacteria and Firmicutes, more represented in Linosa. At the class level, Bacteroidia were more abundant in both Campania nests. Clostridia were more abundant in two samples, one from Linosa nest 2 and one from Campania nest 2 (Fig 5B). At the family level (Fig 5D), each nest was characterized by a different percentage of bacterial strains: *Nitriliruptoraceae*, *Streptococcaceae* and *Planococcaceae* were more abundant in the eggshells of Linosa nest 1, whereas *Rhizobiaceae* and *Flavobacteriaceae* in the Linosa nest 2. *Cellulomonadaceae*, *Alcanivoracaceae* and *Kiloniellaceae* were mainly present in both Campania nests and one sample of Linosa nest 2. Two eggshells (ES_L2_3 and ES_N1_1) contained a large abundance of *Vibrionaceae*, around 30 and 10%, respectively.

ES_L1_1, ES_L2_1 and ES_L2_2 were from fragments of eggshells found in the nest chambers (indicated with an F), and the other samples were derived from the unhatched eggs.

### Microbial composition of the internal content

**Fig 6:**
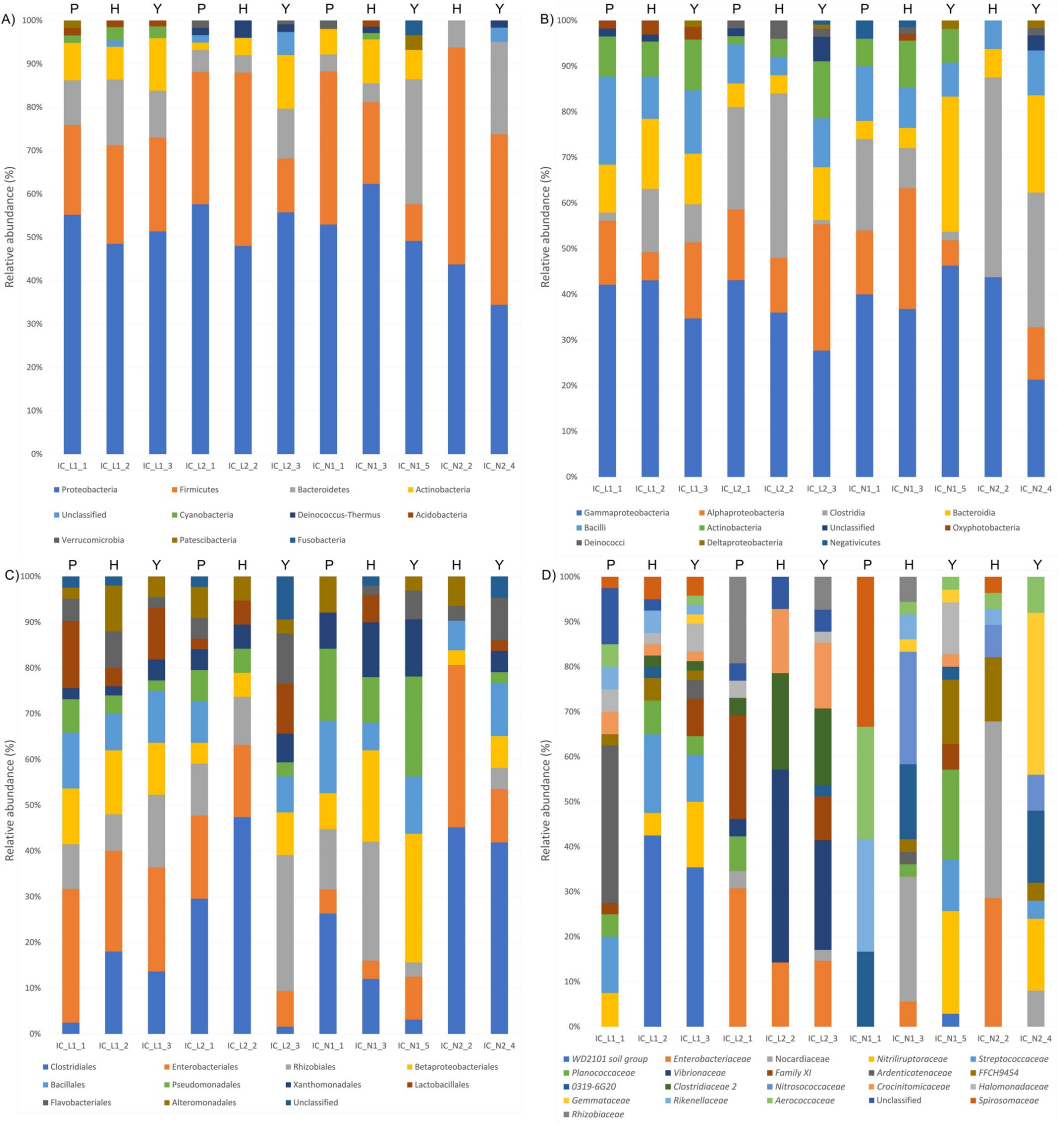
Percentage of the main 10 phyla (A), 10 classes (B), 10 orders (C) and 20 families (D) found in samples of internal content, collected from Linosa 2018 (indicated as IC_L1), Linosa 2019 (indicated as IC_L2), Ascea Marina (indicated as IC_N1) and Eboli (indicated as IC_N2). IC_L1_1, IC_L2_1 and IC_N1_1 were from pipping sea turtles (indicated with a P), IC_L1_2, IC_L2_2, IC_N1_3 and IC_N2_2 were from hatchlings (indicated with an H), the other samples derived from the yolk collected from the unhatched eggs (indicated with a Y).

Proteobacteria with Gammaproteobacteria and Alphaproteobacteria were shown as the dominant phyla accounting for more than 35% of each sample of internal content (Fig 6A). Firmicutes, Bacteroidetes and Actinobacteria represented the main phyla in the internal content of pipping, hatchling and yolk found in all the nests, but two samples of a nest (IC_N2_2 and IC_N2_4) that did not contain any Actinobacteria. Cyanobacteria with the class of Oxyphotobacteria were found in the internal content of all the samples collected from Linosa nest 1 (Fig 6A and 6B). The Clostridia class was relatively abundant in pipping and hatchling of Linosa nest 2 (IC_L2_1 and IC_L2_2), in pipping and hatchling of Campania nests (IC_N1_1 and IC_N2_2) and in one yolk collected from Campania nest 2 (IC_N2_4). When families were analysed, in the cases of a hatchling (IC_L1_2) and a yolk (IC_L1_3) of the same nest Linosa 1, *WD2101 soil group* was found to account for 42.5 and 35.4% respectively (Fig 6D). In all the three samples of Linosa 1, *Streptococcaceae* accounted for 12.5, 17.5, and 10.5% and *Nitriliruptoraceae* for 7.5, 5 and 14.6%, while in all the three samples of Linosa 2, *Enterobacteriaceae* accounted for 30.8, 14.3 and 14.6% and *Vibrionaceae* for 3.9, 42.9 and 24.4%. No specific microbial signatures were found in Campania nests.

## Discussion

In this study, we described for the first time the microbial community of the loggerhead sea turtle *Caretta caretta* nests in two different areas of the Mediterranean Sea, Linosa and Campania (Italy).

To the best of our knowledge, this is the first study describing the bacterial content of eggs and sand collected from the same nests, employing next-generation sequencing of the 16S rDNA gene applied to three main types of samples, internal content, eggshells, and sand. This survey showed that, at the phylum level, the microbial composition of sand samples was similar, yet, each sample showed a specific microbial signature, which depended on the sampling site. This aspect could be ascribed to both the substrate (volcanic *vs* calcareous sand) and the environmental conditions (incubation, temperature, and humidity) of the nest chambers since the chemical/mineralogical composition of the sand could favour colonization by specific bacterial strains [28,47,48].

Our findings highlighted that the sand microbiota features a more substantial number of families than both eggshells and internal contents. Similar conclusions were drawn for the olive ridley sea turtle *Lepidochelys olivacea* in Costa Rica, in the case of *Chelonia mydas*, and hawksbill turtles *Eretmochelys imbricata* in Peninsular Malaysia [48,49]. The low number of families found inside the yolk could be due to either a “filtering” activity of the eggshell or the antimicrobial properties of the albumen proteins. The shell and its membranes of the sea turtle eggs could provide a selective physical barrier to microbial invasion [50]; besides, egg albumen and the mucus secreted from the cloaca during oviposition could act as biological and chemical barriers toward specific taxa [51–53]. Additionally, eggs harboured distinct microbial communities with respect to sand and eggshells, suggesting that oviparous vertebrates may acquire pioneer gut microbiota of maternal origin *in ovo*, as reported in oviparous birds and lizards [14]. In this study, Firmicutes and Bacteroidetes were found more abundant in the internal contents than in the sand and eggshells; thus, internal contents could represent a more suitable environment than sand for the growth of these bacterial phyla, being obligate anaerobes. Relative abundances of Actinobacteria in ES and Sn were quite similar (12%), while IC samples contained Actinobacteria albeit with a low relative abundance (6.6%). Actinobacteria are known as soil bacteria and prolific producers of biologically active secondary metabolites; therefore, they could have a role in inhibiting bacterial and fungal growth during egg development, as reported by other authors [54–57].

Acidobacteria were abundant in the sand of N1 and N2 beaches and scarce in the ES and IC of both nests, strongly indicating an active role of eggshells in selectively permitting the entrance of specific microbial taxa, the difficulty of some taxa to colonizing the internal egg content, or the inhibition of bacterial strains by low-molecular-weight proteins isolated from marine turtle egg white [58–60]. Although Acidobacteria represents one of the most widespread and metabolically diverse phyla [61], its ecological role has not been elucidated yet. Different anthropogenic factors and mineralogical compositions of the sand could influence the microbial flora of the nesting beaches. These aspects could justify the absence of Acidobacteria in the Linosa beach, which is volcanic compared to the calcareous ones in Campania. In addition, the anthropogenic impact could be content in the Linosa beach since it belongs to a Marine Protected Area which limits human activities yearly (www.ampisolepelagie.it).

Our study demonstrated that the internal content of eggs contains a microbiota comparable but not identical to that featuring eggshells. The microbial colonization of the eggs could occur during the egg passage through the maternal cloaca or oviduct or during nesting and oviposition. A delay in the microbial acquisition of specific species could make animals more sensitive to pathogen attacks, influencing egg hatching. However, we cannot surmise if bacterial colonization is a good sign for hatching success since control eggs were not analysed. Microbial colonization could be part of a development process in which resident bacteria of the ovary/oviduct, cloaca, or sand pass through the eggshells. However, bacteria could act as pathogens hampering egg development. Indeed, other studies reported the isolation of various Gram-negative -positive pathogen species in fluid samples from the egg interior of unhatched *C*. *caretta* sea turtle eggs [31,34,35]. In these studies, pathogens such as *Pseudomonas aeruginosa*, *Serratia marcescens*, *Salmonella*, *Aeromonas*, *Citrobacter*, as well as eukaryotic ones (i.e., *Fusarium* and *Aspergillus* fungal strains) were detected [35,48].

Egg microbiota could be of fundamental importance for the survival of the species as dysbiosis linked to exogenous factors (chemical pollution, pathogens of various kinds released by human activities, plastics, etc.) can have a role in health status. Many stressors could disturb the nest chamber, such as sand pollutants, weather events (e.g., tides, rains, wind), or predators, such as the ghost crab *Ocypode cursor* [62].

As far as we know, the microbiota analysis of eggshells was carried out on the sea turtle *Eretmochelys imbricata* which is mainly dominated by Proteobacteria, Actinobacteria, Firmicutes, and Bacteroidetes [63], as we found in the present study. Proteobacteria is the predominant phylum in the microbiota of the nesting sea turtles, as recently described [19]. We could surmise that the high abundance of Proteobacteria in the ES could depend on maternal influence and environmental components (such as the mineralogical substrate of the beach, seawater, and exogenous bacterial source). Conversely, Firmicutes and Bacteroidetes are less abundant in the sand and seawater [64], thus suggesting an influence of the mother in providing eggs with these bacteria during the passage through the oviduct or cloaca.

This study highlights that a deep evaluation of the nesting beach at diverse levels (i.e., sand, water, terrestrial nutrients source, anthropic activities, pollutants, etc.), alongside the microbial components of sea turtle nest, could provide insights to delineate conservation protocols aimed at protecting the loggerhead sea turtle *C*. *caretta*. Moreover, in light of these results, a special effort is needed in a constant and shared monitoring activity of the Mediterranean loggerhead sea turtle population to support efficient measures to preserve all coastal areas potentially chosen as nesting beaches. Even if we are in the infancy of this kind of study, the microbial profile associated with environmental parameters could be a good monitoring tool to establish vulnerable nesting areas to preserve.

## Supporting information

S1 Fig Rarefaction curves of the samples analysed in this study. (TIF)

S2 Fig The expression heat map is based on the detected families of all studied samples, generated by the “complete linkage” calculation and Spearman Rank correlation. (TIFF)

S1 Table Total number of ASVs resulting from the QIIME2 pipeline. (DOCX)
