# Supplementary figures and images for: Unveiling the egg microbiota of the loggerhead sea turtle Caretta caretta in nesting beaches of the Mediterranean Sea
Source: PLoS One. 2022 May 26;17(5):e0268345. doi: 10.1371/journal.pone.0268345 (PMC9135217; doi:10.1371/journal.pone.0268345)

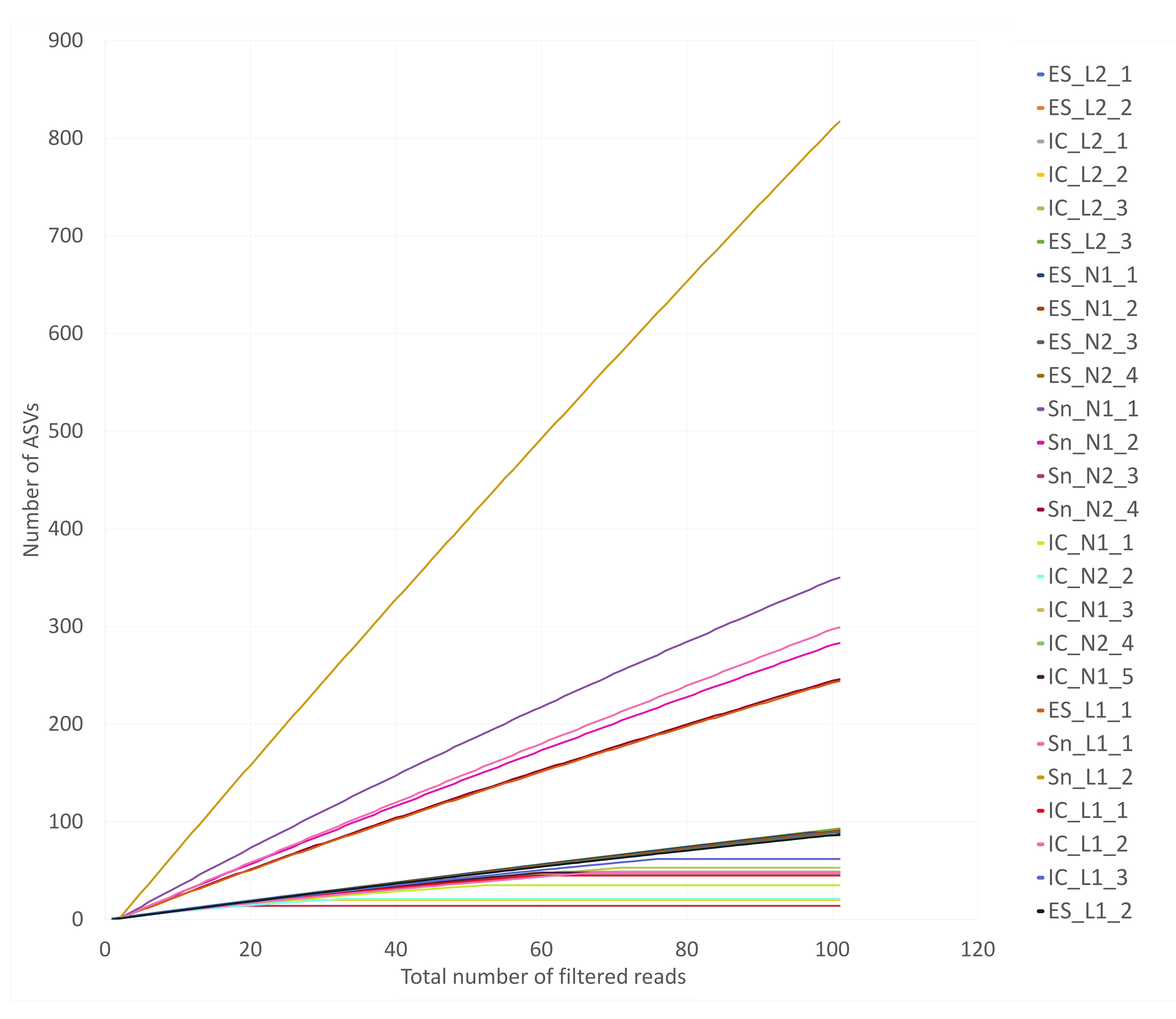

Supplement: S1 Fig — (TIF) [file pone.0268345.s001.tif]

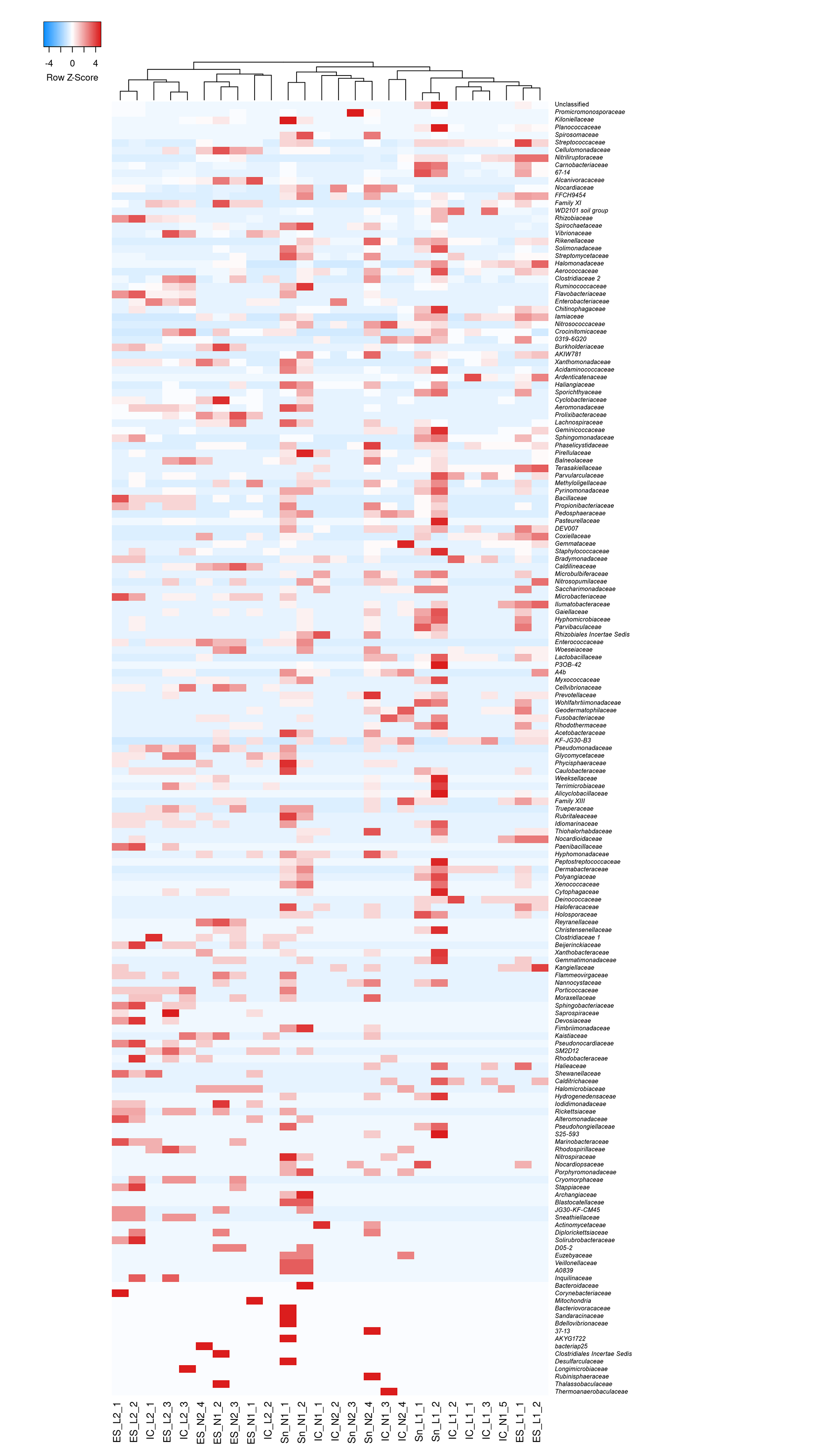

Supplement: S2 Fig — (TIFF) [file pone.0268345.s002.tiff]
