# Supplementary material for: Unveiling the egg microbiota of the loggerhead sea turtle Caretta caretta in nesting beaches of the Mediterranean Sea
Source: PLoS One. 2022 May 26;17(5):e0268345. doi: 10.1371/journal.pone.0268345 (PMC9135217; doi:10.1371/journal.pone.0268345)
Supplement: S1 Table — (DOCX) [file pone.0268345.s003.docx]

**Table S1.** Total number of ASVs resulting from the QIIME2 pipeline.

| **Sample** | **Total Reads** | **Filtered Reads** | **Merged Reads** | **Chimeras** | **ASVs** |
| --- | --- | --- | --- | --- | --- |
| ES_L1_1 | 69280 | 48138 | 45864 | 1787 | 283 |
| ES_L1_2 | 90186 | 64261 | 62501 | 2585 | 166 |
| ES_L2_1 | 70422 | 57604 | 53043 | 11317 | 105 |
| ES_L2_2 | 61580 | 51164 | 47693 | 5924 | 143 |
| ES_L2_3 | 68706 | 55038 | 51519 | 11418 | 128 |
| ES_N1_1 | 59665 | 48225 | 45921 | 10253 | 96 |
| ES_N1_2 | 63976 | 51698 | 47294 | 12346 | 191 |
| ES_N2_3 | 56702 | 45062 | 42241 | 4533 | 132 |
| ES_N2_4 | 67334 | 53669 | 48527 | 13161 | 98 |
| IC_L1_1 | 62000 | 38332 | 37362 | 918 | 58 |
| IC_L1_2 | 114904 | 82549 | 81445 | 4734 | 66 |
| IC_L1_3 | 45105 | 32586 | 32171 | 232 | 75 |
| IC_L2_1 | 86447 | 70423 | 68445 | 15892 | 59 |
| IC_L2_2 | 73247 | 60396 | 58400 | 16963 | 25 |
| IC_L2_3 | 60439 | 48288 | 46821 | 3340 | 114 |
| IC_N1_1 | 57356 | 47058 | 46634 | 291 | 51 |
| IC_N1_3 | 47315 | 38005 | 37503 | 126 | 32 |
| IC_N1_5 | 55853 | 45302 | 44338 | 516 | 70 |
| IC_N2_2 | 76944 | 64088 | 61096 | 21676 | 62 |
| IC_N2_4 | 87930 | 71898 | 68597 | 18450 | 59 |
| Sn_L1_1 | 30692 | 20836 | 19605 | 55 | 318 |
| Sn_L1_2 | 69816 | 49471 | 46766 | 389 | 877 |
| Sn_N1_1 | 56900 | 44248 | 43503 | 351 | 410 |
| Sn_N1_2 | 50201 | 31520 | 24655 | 1817 | 315 |
| Sn_N2_3 | 18254 | 1137 | 497 | 0 | 335 |
| Sn_N2_4 | 81727 | 48961 | 35604 | 5025 | 289 |
